# Beneficial effects of inhaled NO on apoptotic pneumocytes in pulmonary thromboembolism model

**DOI:** 10.1186/1742-4682-11-36

**Published:** 2014-08-10

**Authors:** Chaosheng Deng, Minxia Yang, Qichang Lin, Yuanhua Yang, Zhenguo Zhai, Kaixiong Liu, Haibo Ding, Xiaoming Cao, Zhihua Huang, Lina Zhang, Jianming Zhao

**Affiliations:** 1 Department of Respiratory Disease, First Affiliated Hospital of Fujian Medical University, 350005 Fuzhou, Fujian Province, China; 2 Division of Respiratory and Critical Care Medicine, Beijing Chaoyang Hospital, Capital Medical University, 100020 Beijing, China

**Keywords:** Lung ischemia-reperfusion injury, Pulmonary thromboembolism, Apoptotic pneumocytes, Inhaled nitric oxide

## Abstract

**Background:**

Lung ischemia–reperfusion injury (LIRI) may occur in the region of the affected lung after reperfusion therapy. Inhaled NO may be useful in treating acute and chronic pulmonary thromboembolism (PTE) due to the biological effect property of NO.

**Methods:**

A PTE canine model was established through selectively embolizing blood clots to an intended right lower lobar pulmonary artery. PaO2/FiO2, the mPAP and PVR were investigated at the time points of 2, 4, 6 hours after inhaled NO. Masson’s trichrome stain, apoptotic pneumocytes and lung sample ultrastructure were also investigated among different groups.

**Results:**

The PaO2/FiO2 in the Inhaled NO group increased significantly when compared with the Reperfusion group at time points of 4 and 6 hours after reperfusion, mPAP decreased significantly at point of 2 hours and the PVR decreased significantly at point of 6 hours after reperfusion. The amounts of apoptotic type II pneumocytes in the lower lobar lung have negative correlation trend with the arterial blood PaO2/FiO2 in Reperfusion group and Inhaled NO group. Inhaled nitric oxide given at 20 ppm for 6 hours can significantly alleviate the LIRI in the model.

**Conclusions:**

Dramatic physiological improvements are seen during the therapeutic use of inhaled NO in pulmonary thromboembolism canine model. Inhaled NO may be useful in treating LIRI in acute or chronic PTE by alleviating apoptotic type II pneumocytes. This potential application warrants further investigation.

## Introduction

Pulmonary thromboembolism (PTE) has been experienced by 60% of patients who die in the hospital and it is currently the third most common cause of death in hospitalized patients [1].

Lung ischemia–reperfusion injury (LIRI) may occur in the region of the affected lung after the thrombolytic therapy, pulmonary embolectomy, pulmonary suction thrombectomy [2-4], or the alternative interventional strategy of balloon pulmonary angioplasty (BPA) for chronic thromboembolic pulmonary hypertension (CTEPH) [5].Clinically, LIRI is characterized by reperfusion pulmonary edema (RPE) developing within 48 h post-operation (e.g., thromboendarterectomy, lung transplant, etc.). The patients are subjected to prolonged intubation for mechanical ventilation, ventilator-associated infections, longer stays in the intensive care unit (ICU), and early postoperative mortality [6-8].

Programmed cell death (apoptosis) appears to be a significant type of cell loss in human lungs after transplantation. A number of studies in humans demonstrate that reperfusion after ischemia induces apoptosis in more than 20% of parenchymal lung cells (mainly type II pneumocytes) after lung transplantation [9,10]. This cell loss might be responsible for severe organ dysfunction [10,11]. The mechanisms related to the LIRI in PTE, especially apoptosis of the pneumocytes, are less deeply understood as that of lung transplantation [12]. Because of organization and recanalization channels within the chronic thrombus, the ischemic changes of chronic PTE may be less when compared to the ischemic changes brought about by deliberate/experimental ligation or other inert blockage. At present, little is known about the relation between LIRI and the induction of apoptosis on pneumocytes in acute or chronic PTE.

The treatment with inhaled nitric oxide(NO) has been advocated to improve RPE after pulmonary suction thrombectomy and pulmonary thromboendarterectomy for chronic PTE [13-15]. However, this treatment does not work in all patients. Studies examining the inhalation delivery of NO in experimental and clinical applications have shown discrepant results; some show to mitigate LIRI including its antioxidant properties and its cytoprotective abilities including attenuating apopotosis [16-18], whereas others demonstrate detrimental or neutral effects [19,20] and the detailed mechanisms remain to be understood.

We have successfully established a reproducible modified experimental canine PTE model with autologous cylinder blood clots selectively embolized into any intended lobar artery with guidance of Swan-Ganz float catheter under X-ray fluoroscopy. This model may mimic the pathological changes of chronic PTE and the location of thrombus is similar to proximal type of CTEPH [21]. In PTE, the pulmonary lower lobar artery is more commonly involved due to a more extensive circulation [22]. Therefore, we aimed at precisely embolizing the right lower pulmonary lobar artery. Two weeks later after selective embolization, we performed embolectomy for reperfusion as to examine the LIRI changes, especially apoptotic pneumocytes.

## Results

**Figure 1:**
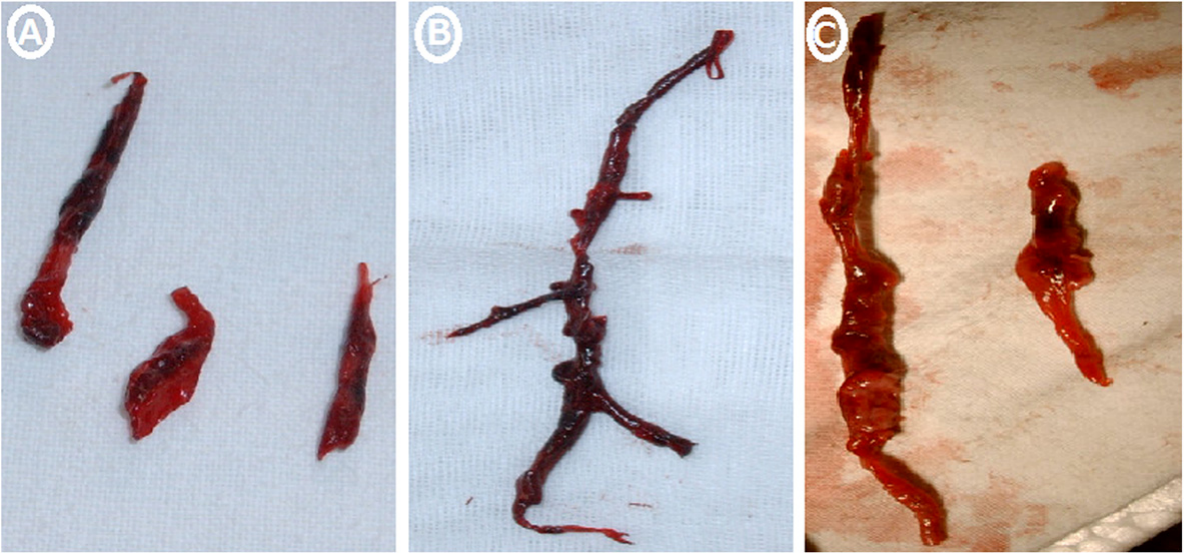
**The thrombus is shown after embolectomy from a lower pulmonary lobar artery.** The thrombi were either multiple fragmentary segments **(Figure** 1**A, C)** or a complete elongated strip with multiple pink granulation-like protrusions and multiple branches corresponding to the pulmonary artery branches **(Figure **1**B)**.

**Figure 2:**
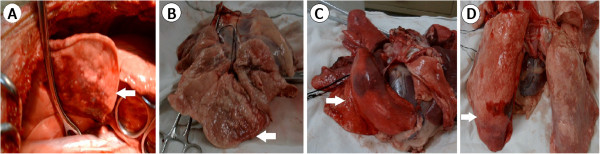
**The gross appearance of the lung in the four groups. A**. The Sham group showed pink lung with a little congestion (white arrow). **B**. The Ischemia group showed a reddish-gray lung with some collapsed appearance (white arrow). **C**. The Reperfusion group showed a reddish lung with congested and swollen appearance (white arrow) with some dark red appearance indicating bleeding. **D**. The Inhaled NO group showed a somewhat similar but less congested and swollen lung appearance as the reperfusion group (white arrow).

1. Three main types of reddish brown thrombus with irregular surface were shown after embolectomy from lower pulmonary lobar artery. The thrombi were either multiple fragmentary segments (Figure 1A, C) or a complete elongated strip with multiple pink granulation-like protrusions and multiple branches corresponding to the pulmonary artery configuration (Figure 1B).2. The macroscopic pathology of the lung in the four groups was shown in Figure 2. Sham group showed the pink lung appearance with a little congestion (Figure 2A). Ischemia group showed reddish-greyish color lung with some collapsed appearance (Figure 2B). Reperfusion group showed reddish lung with congestion and swelling (Figure 2C), some parts of which with dark red areas that indicate bleeding. Inhaled NO group showed pink color lung with congestion and swelling, some small parts of which with bright red to dark red areas that indicate bleeding (Figure 2D). The congested and swollen lung appearance shown in inhaled NO group was lighter than that in reperfusion group.

3. The arterial blood PaO2/FiO2 and hemodynamic parameters (HR, mPAP, CO, PVR) in four groups are shown in Table 1.

**Table 1:** **HR, arterial blood gases and hemodynamic parameters at each time point after surgical procedures in four groups are shown (n = 6 in each group)**$\left(\bar{x}\pm s\right)$

	**0 h**	**2 h**	**4 h**	**6 h**
HR (beats/min)				
Group 1	152 ± 5	155 ± 7	154 ± 8	157 ± 8
Group 2	155 ± 7	158 ± 6	156 ± 9	160 ± 7
Group 3	155 ± 5	162 ± 5^*#^	168 ± 9^*#Δ^	175 ± 8^*^
Group 4	154 ± 6	160 ± 6	168 ± 8^*Δ^	167 ± 7^*^
pH				
Group 1	7.32 ± 0.06	7.33 ± 0.03	7.29 ± 0.10	7.31 ± 0.01
Group 2	7.25 ± 0.04	7.26 ± 0.04	7.24 ± 0.03	7.21 ± 0.01
Group 3	7.25 ± 0.11^#^	7.11 ± 0.18	7.23 ± 0.05	7.15 ± 0.14^#^
Group 4	7.27 ± 0.05	7.18 ± 0.19	7.13 ± 0.11^#^	7.15 ± 0.20
PaCO_2_ (mmHg)				
Group 1	36.88 ± 6.06	41.52 ± 12.41	40.18 ± 13.86	40.84 ± 10.62
Group 2	38.88 ± 6.66	42.52 ± 12.41	42.18 ± 10.86	44.84 ± 7.62
Group 3	41.43 ± 9.51	56.77 ± 22.75	49.15 ± 14.03	47.48 ± 14.15
Group 4	38.88 ± 6.66	48.52 ± 22.41	45.18 ± 13.86	47.84 ± 17.62
PaO2/FiO2(mmHg)				
Group 1	484.42 ± 28.34	475.50 ± 20.80	543.45 ± 61.56	423.20 ± 23.20
Group 2	430.95 ± 28.37	428.75 ± 37.50	431.25 ± 39.24◆	435.00 ± 31.62◆
Group 3	410.40 ± 28.36	335.55 ± 29.29^*#Δ^	287.90 ± 54.84^*#Δ^	292.83 ± 60.34^*#Δ^
Group 4	441.43 ± 24.26	371.87 ± 20.35^*#^	368.83 ± 55.29^*#Δ^◆	380.63 ± 56.83◆
MPAP (mmHg)				
Group 1	14 ± 1	14 ± 1	13 ± 3	12 ± 1
Group 2	16 ± 2	15 ± 2	14 ± 1	15 ± 2
Group 3	17 ± 3	24 ± 4^*#Δ^	22 ± 4^#Δ^	18 ± 4^#§^
Group 4	17 ± 4	19 ± 3^#Δ^◆	20 ± 2^#Δ^	15 ± 2^#§^
CO(l/min)				
Group 1	2.95 ± 0.32	2.84 ± 0.24	2.84 ± 0.69	2.86 ± 0.53
Group 2	2.60 ± 1.00	2.62 ± 1.04	2.62 ± 1.08	2.73 ± 1.03
Group 3	2.88 ± 1.04	2.60 ± 1.14	2.71 ± 1.28	2.63 ± 1.19
Group 4	3.01 ± 0.64	2.62 ± 0.26	3.17 ± 0.54	3.20 ± 0.55
PVR(dyne.s.cm^-5^)				
Group 1	164.24 ± 45.61	200.94 ± 59.54	140.46 ± 55.84	171.59 ± 38.72
Group 2	301.25 ± 96.77^#^	253.68 ± 99.09	299.52 ± 92.64^#^	271.66 ± 93.89^#^
Group 3	373.20 ± 89.09^#^	601.01 ± 92.33^*#Δ^	435.88 ± 99.63^#^	327.09 ± 96.66^#§^
Group 4	335.88 ± 63.46^#^	474.67 ± 87.91^*#^	335.93 ± 43.84^#^	239.86 ± 53.26^*^◆^§^

Note: Group 1: Sham group, Group 2: Ischemia group, Group 3: Reperfusion group, Group 4: Inhaled NO group. * and § *P* < 0.05 for the comparison with the parameters recorded at the time point before reperfusion and 2 hours after reperfusion within the same group, respectively. At the same time point among different groups,#*P* < 0.05 for the comparison with the Sham group,Δ *P* < 0.05 for the comparison with the Ischemia group,◆ *P* < 0.05 for the comparison with the Reperfusion group.

3.2 The parameters at each time point after surgical procedures between the different groups were compared.

HR at the time point of 4 hours after embolectomy in Reperfusion group was significantly faster than the Sham group (168 ± 9 beats/min vs. 154 ± 8 beats/min, *P* < 0.05) and the Ischemia group (168 ± 9 beats/min *vs.* 156 ± 9 beats/min, *P* < 0.05).

The PaO2/FiO2 in the Reperfusion group decreased significantly at point 4 and 6 hours after embolectomy when compared with the Ischemia group (287.90 ± 54.84 mmHg *vs.* 431.25 ± 39.24 mmHg) and (292.83 ± 60.34 mmHg *vs.* 435.00 ± 31.62 mmHg), respectively (both *P* < 0.05). The PaO2/FiO2 in the Inhaled NO group increased significantly when compared with the Reperfusion group at points 4 and 6 hours (368.83 ± 55.29 mmHg *vs.* 287.90 ± 54.84 mmHg) and (380.63 ± 56.83 mmHg *vs.* 292.83 ± 60.34 mmHg(both *P* < 0.05).

The mPAP in Reperfusion group increased significantly at point 2 and 4 hours after reperfusion when compared with the Ischemia group (24 ± 4 mmHg *vs.* 15 ± 2 mmHg) and (22 ± 4 mmHg *vs.* 14 ± 1 mmHg), respectively (both *P* < 0.05). In the Inhaled NO group, when compared with the Reperfusion group, mPAP decreased significantly at point 2 hours after reperfusion (19 ± 3 mmHg *vs.* 24 ± 4 mmHg, *P* < 0.05); while at points 4 and 6 hours after reperfusion, a decreasing mPAP trend was noted but without significant difference (*P* > 0.05).

PVR in Reperfusion group increased significantly at point 2 hours after embolectomy when compared with Ischemia group (601.01 ± 92.33 dyne.s.cm-5 *vs.* 253.68 ± 99.09 dyne.s.cm-5, *P* < 0.05). In Inhaled NO group, at time point of 6 hours after reperfusion with inhaled NO, the PVR decreased significantly when compared with that in the Reperfusion group (239.86 ± 53.26 *vs. *327.09 ± 96.66, *P* < 0.05).

3.1 The parameters at different time points after reperfusion within the same group were compared.

In Sham group and Ischemia group, there was no significant difference on the PaO2/FiO2 and hemodynamic parameters at different time points after surgical procedures (*P* > 0.05).

In the Reperfusion group, the heart rates at the time points of 2, 4, 6 hours after embolectomy were significantly faster than baseline, most obviously at the time point of 6 hours after reperfusion (175 ± 8 beats/min *vs.* 155 ± 5 beats/min, *P* <0.05). The PaO2/FiO2 decreased significantly at the time points of 2, 4, 6 hours after reperfusion than that of baseline, most obviously at point 4 hours after reperfusion (287.90 ± 54.84 mmHg *vs.* 410.40 ± 28.36 mmHg, *P* <0.05). The mPAP increased significantly at point 2 hours after reperfusion (24 ± 4 mmgHg *vs.* 17 ± 3 mmHg, *P* <0.05). At the time point of 4 hours after reperfusion, mPAP increased but without significant difference (*P* > 0.05) and decreased to the baseline level at point of 6 hours after reperfusion. The PVR increased significantly at time point of 2 hours after reperfusion when compared with that of baseline (601.01 ± 92.33 dyne.s.cm-5 *vs.* 373.20 ± 89.09 dyne.s.cm-5, *P* <0.05), then decreased gradually to the baseline level.

In the Inhaled NO group, PaO2/FiO2 decreased significantly at the time points of 2, 4 hours after reperfusion than that of baseline, most obviously at point 4 hours after reperfusion (368.83 ± 55.29 mmHg *vs.* 441.43 ± 24.26 mmHg, *P* <0.05) and increased gradually at the time point of 6 hours after reperfusion without significant difference when compared with the baseline (380.63 ± 56.83 mmHg *vs.* 441.43 ± 24.26 mmHg, *P* > 0.05). The mPAP increased at time point of 2 and 4 hours after reperfusion while at the point of 6 hours it decreased to the level much lower than the point of 4 hours (15 ± 2 mmHg *vs.* 20 ± 2 mmHg, *P* <0.05).

**Figure 3:**
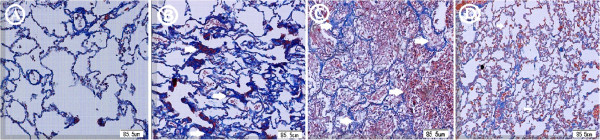
**Pathology stained by Masson's trichrome stain was shown in different groups. A**. The alveolar structure with some exudation in the right lower lung was shown in the Sham group. **B**. Some collapsed alveolar structures, atelectasis with thickened alveolar septa (white arrow), collagen fibers, and a few exudative cells in the alveolar space were shown in the Ischemia group. **C**. Incomplete and destructed alveolar structure with a large number of exudative cells and exudation (white arrow) were seen within the alveolar spaces in the Reperfusion group. **D**. Alveolar structures with exudative cells and exudation were shown in the Inhaled NO group, but less when compared with the Reperfusion group (white arrow).

4. Pathology stained by Masson’s trichrome stain was shown in different groups.The alveolar structure with some exudation in the right lower lung of the Sham group was shown in Figure 3A. Some collapsed alveolar structures, thickened alveolar septa, and collagen fibers stained blue and a few exudative cells in the alveolar space were shown in the Ischemia group (Figure 3B). Incomplete and destructed alveolar structure with a large number of exudative cells and exudation were seen within the alveolar spaces in the Reperfusion group (Figure 3C). Similar incomplete and destructed alveolar structures with exudative cells and exudation were shown in the Inhaled NO group, but much less when compared with the Reperfusion group (Figure 3D).

5. Apoptotic pneumocytes among different groups. and the correlations between the amounts of apoptotic type II pneumocytes and PaO2/FiO2.

**Figure 4:**
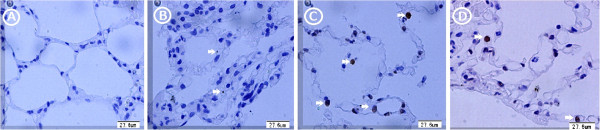
**The apoptotic pneumocytes were observed in the segment distal the clot. A**. There were no apoptotic cells detected on slides in Sham group. **B**. In the Ischemia group, the slides revealed some apoptotic pneumocytes shown by TUNEL (white arrow). **C**. In the Reperfusion group, the number of apoptotic pneumocytes was much more than Ischemia group (white arrow). **D**. In the Inhaled NO group, there were also many apoptotic pneumocytes (white arrow).

The apoptotic pneumocytes were observed in the segment distal the clot. No apoptotic cell was detected in the Sham group (Figure 4A). In the Ischemia group, some apoptotic pneumocytes (1 ± 1 pneumocytes/5 fields, 400’) were revealed by TUNEL method (Figure 4B). Six hours after surgery, the number of apoptotic pneumocyte in Reperfusion group was more than that of the Ischemia group (5 ± 1 pneumocytes/5 fields *vs.* 1 ± 1 pneumocytes/5 fields, Figure 4C). In the Inhaled NO group, the number of apoptotic pneumocyte was also much more than the Ischemia group (5 ± 2 pneumocytes/5 fields *vs.* 1 ± 1 pneumocytes/5 fields, *P* < 0.05, Figure 4D) The amounts of apoptotic type II pneumocytes in the lower lobar lung have negative correlation trend with the arterial blood PaO2/FiO2 in Reperfusion group and Inhaled NO group.

**Figure 5:**
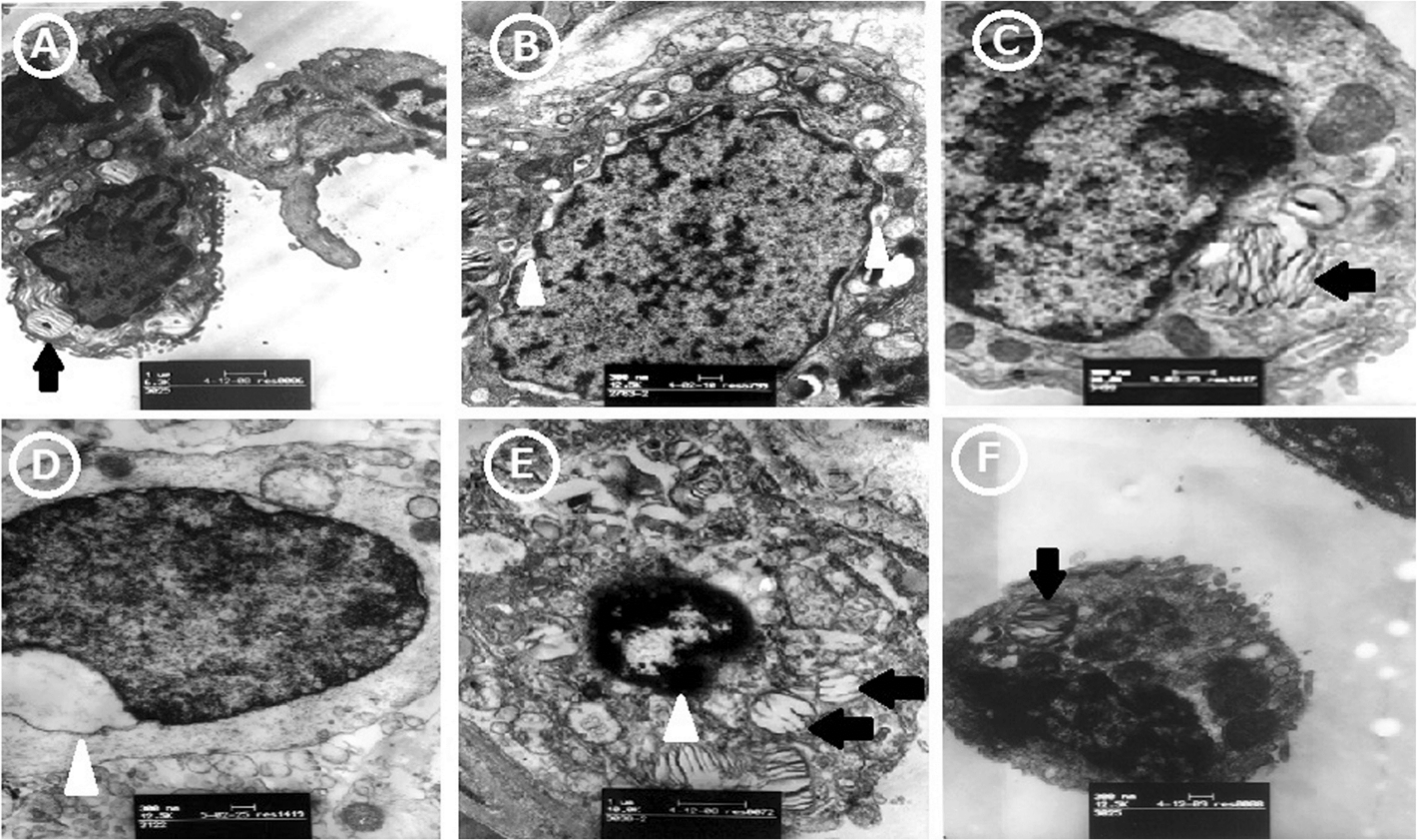
**Lung sample ultrastructure was evaluated by electron microscopy. A**. The ultrastructural architecture of type II pneumocytes as evaluated by Electron Microscopy with normal lamellar bodies, mitochondria, tight junction, and microvilli was normal in Sham group. **B**. The perinuclear gap in the type II pneumocytes was widened and showed early stage of apoptosis in the Ischemia group (white arrow head). **C**, **D**. Swelling of lamellar bodies (black arrow) in some type II pneumocytes and nuclear membrane blebbing (white arrow head) were shown in the Inhaled NO group. **E**. Apoptosis was indicated by more extensive nuclear shrinkage, chromatin condensation and margination (white arrow head), whereas plasma membrane and intracellular organelles such as lamellar bodies showed degenerative changes (black arrow) but had an intact surface in the Reperfusion group. **F**. The type II pneumocyte with lamellar bodies swelling (black arrow), cytoplasmic vacuolation, and irregular nuclear shed into the alveolar space in Reperfusion group.

6. Lung sample ultrastructure was evaluated by electron microscopy.Lung ultrastructural architecture was normal in the Sham group and type II pneumocytes did not show evidence of apoptosis with normal lamellar bodies, mitochondria, tight junction and microvilli (Figure 5A). The perinuclear gap in the type II pneumocytes was widened and showed early stage apoptosis in the Ischemia group (Figure 5B). Swelling of lamellar bodies in some type II pneumocytes and nuclear membrane blebbing were shown in the Inhaled NO group (Figure 5C, D). Apoptosis was indicated by more extensive nuclear shrinkage, chromatin condensation and margination, whereas plasma membrane and intracellular organelles such as lamellar bodies showed degenerative changes but had intact surface in the Reperfusion group (Figure 5E). Furthermore, the type II pneumocyte had lamellar bodies swelling, cytoplasmic vacuolation, and irregular nuclei shed into the alveolar space in Reperfusion group (Figure 5 F).

## Discussion

Previously, we have successfully established a modified canine PTE model. This model has mimicked the pathological changes of chronic PTE. Due to a precise embolization into the intended right lower pulmonary artery, it is convenient for us to perform embolectomy for investigating the effects of the LIRI in the model. The three types of reddish brown thrombus enucleated by embolectomy from the involved pulmonary lobar artery revealed the irregular surface with multiple pink granulation-like protrusions and multiple branches corresponding to the lobar artery branches. Generally, during ischemia-reperfusion injury in systemic vascular beds, the vascular response appears to occur in at least two phases: (1) ischemia, which is associated with lack of oxygen, cell damage, and activation of cytotoxic enzymes, and (2) reperfusion, which is associated with formation of reactive oxygen intermediates, platelet and neutrophil activation, endothelial cell injury, increased vascular permeability, cytokine activity, and complement activation [23]. The multiple recanalization channels within the organized thrombus, which are associated with the persistent period of embolism, is significantly correlated to the reduction in PVR [24]. As demonstrated by macropathology and Masson’s trichrome stain in the ischemia group, LIRI results in atelectasis, fibrosis and moderate inflammation characterized by a ‘chronic’ fibroproliferative state, including infiltration, collagen deposition and pulmonary remodeling [25]. The microenvironment provided by the unresolved clot and inflammatory cells may stimulate this erroneous cell proliferation and injure cells [26,27]. Hypoxic conditions can enhance the production of inducible nitric oxide synthetase (NOS) that may increase oxidant radical byproducts, such as the peroxynitrite anion, and lead to reactive oxygen species (ROS)-type cellular injury [28]. ROS have diverse actions on pulmonary tissue including smooth muscle contraction, interaction with redox enzymes, cell proliferation, and gene transcription [29]. Apoptosis is a genetically controlled programmed cell death resulting from activation of the intrinsic (mitochondrial) and extrinsic (death receptor) pathways; it can be triggered by mechanical injury and exposure to certain environmental conditions such as hypoxic conditions [30]. Governing such pathways to the lung apoptosis after ischemia–reperfusion can open new vistas for reducing the severity of LIRI [31]. Apoptosis is characterized morphologically by nuclear condensation and shrinkage followed by fragmentation of nuclear chromatin without typical inflammation, which have been showed by TUNEL in our study.

With re-establishment of blood flow, the injury elicited by reperfusion can be more severe than that caused by ischemia per se; the injury may also result from the combined effects of ischemia and reperfusion [24]. The most important factor complicating the early postoperative period after pulmonary thromboendarterectomy is RPE [32]. This injury is due primarily to mechanisms that cause alveolar epithelial cell damage and increase pulmonary vascular permeability, as have been shown by the Masson’s trichrome stain of the pathological sections. Thus, the injury is associated with impaired gas exchange (exemplified by the decreased PaO2/FiO2) from increased edema formation and surfactant inactivation Carrico index (ratio of arterial PO2 (mmHg)/inspired O2 (fraction)) is the most useful measure of pulmonary oxygenation in clinical practice and stands for the objective index for the oxygenation. Therefore, in our model, the PaO2/FiO2 decreased significantly at the time points of 2, 4, 6 hours after reperfusion than that of baseline as LIRI occurs in patients endarterectomized and reperfused [33]. The accepted theory for reperfusion lung injury is primarily also based on the generation of ROS upon reperfusion, causing cellular damage and apoptosis [30,34]. Even a low number of apoptotic cells at a given time point may indicate a substantial rate of cell loss [35]. A direct correlation between the degree of lung apoptosis after lung ischemia–reperfusion and pulmonary dysfunction remains controversial. When sufficient injury is inflicted on a cell, there are two major types of cell death that can occur: necrosis and apoptosis. Necrosis is a form of irreversible cell death accompanied by the loss of cell membrane integrity and ion pump damage. The clinical manifestations of RPE occurring after interventions for PTE are similar to RPE after lung transplantation [5-8]. Of particular importance after lung transplantation is severe life-threatening graft dysfunction that occurs in up to 30% of patients; this is related to LIRI [36]. A study concluded that a significant number of pneumocytes undergo apoptosis after reperfusion, that the peak in the number of apoptotic pneumocytes occurs as early as 2 hours after reperfusion in the transplanted rat lung [37]. In our experimental model, apoptotic pneumocytes induced by LIRI were much more than those in the Ischemia group. These apoptotic pneumocytes were confirmed by EM ultrastructural evaluation to be mainly type II pneumocytes, with the similar LIRI results from lung transplantation, trauma, and surgical procedures such as cardiopulmonary bypass [11,38].

Inhaled NO may be useful in treating acute and chronic pulmonary embolism due to the biological vasodilatory effect property of NO [13,39,40]. In our experimental model, when inhaled NO at 20 ppm was administered, the PaO2/FiO2 decreased significantly at the time points of 2, 4 hours after reperfusion by embolectomy and, at the time point of 6 hours after reperfusion, increased gradually to the baseline mainly due to the improvement in V/Q matching [3,14,15]. When compared with the Reperfusion group, the PaO2/FiO2 increased significantly after inhaling 20 ppm NO for 4 or 6 hours, one of the mechanisms relating to elevating inducible nitric oxide synthase (iNOS) expression, and NOS activity in the lungs [41]. After inhaling NO for 6 hours, the mPAP decreased to the level much lower than that of the 4 hours and the PVR decreased significantly possibly due to the regional vasodilatation effect of inhaled NO [3,14,15] when compared with that in the Reperfusion group , which are in accordance with the clinical investigations of RPE after pulmonary suction thrombectomy and pulmonary thromboendarterectomy for chronic PTE.

In addition, the amounts of apoptotic type II pneumocytes have negative correlation trend with the arterial blood PaO2/FiO2 because the apoptotic pneumocytes are of insufficience for the surfactant secretion, preventing lung collapse, improving oxygenation. These findings are in accord with a randomized controlled clinical study on PTE, lung transplantation and the mechanisms [30,42]. The mechanisms of injury may involve neutrophil activation, oxygen radicals, cytokines, complement, arachidonic acid derivatives, platelet activating factor [43]. The LIRI can be effectively blunted by the reduction of macrophage-dependent injury by gadolinium while inhaled NO also will attenuate injury by reducing pulmonary hypertension and minimizing neutrophil sequestration [44]. Study has also found that inspired NO promoted the integrity of pulmonary endothelium, increased the vascular density and alleviated the lung histological injury compared to ARDS by augmenting the mRNA expression of Endothelial Progenitor Cells (EPC) surface markers CD34 and CD133 in lung tissue [45].

Studies suggest that inhaled NO can be either protective or toxic to the lung depending on the dose, timing, duration of NO administration, source of NO and the local re-dox environment; these indicate that there is a narrow therapeutic window for nitric oxide applications [16,46,47]. Investigations have shown that the maximum protective effect is achieved with NO concentrations between 10 and 20 ppm [48]. Inhaled NO at doses of 15–20 ppm is routinely given for the first 4 h postoperatively and gradually withdrawn [49].

However, currently, no randomized controlled studies have recommended the routine use of inhaled NO in lung surgery of transplantation or PTE [50]. Therefore, further studies may focus on the molecular interactions among apoptotic factors in LIRI [11,51,52].

## Conclusions

Dramatic physiological improvements are seen during the therapeutic use of inhaled NO in pulmonary thromboembolism canine model. Inhaled NO may be useful in treating LIRI in acute or chronic PTE by alleviating apoptotic type II pneumocytes. This potential application warrants further investigation.

## Materials and methods

### Animals and study design

Animal procedures were approved by the Fujian Medical University Institutional Animal Care and Use committee and all experiments were conducted in accordance with the Guide for the Care and Use of Laboratory Animals.

A total of 24 healthy mongrel canines (weight, 20 ± 1.8 kg) were randomly divided into four groups: In the Sham group (Group 1, n = 6), the procedures were the same as in the other groups, except that, instead of the autologous cylinder blood clots, 0.9% NaCl was infused into the canine right lower pulmonary lobar artery. The remaining 18 canines underwent selective embolization. That is, twenty milliliters of autologous blood extracted from the canine’s saphenous vein using a 20 ml syringe were rapidly injected into three segmental cylinder tubes of pliable medical sterile intravenous transfusion polyvinyl chloride(PVC) with length of 7 cm, inner diameter of 4 mm (the tube was named as tube I) to form the cylinder autologous blood clots at room temperature. Eight hours later, all blood clots were placed into a sterile container with 37°C saline for later use. Then the right external jugular vein was dissected and cannulated with a 7 F sheath. A Swan-Ganz float catheter (Edwards Lifesciences Llc, One Edwards Way Irvine,CA92614) was used to guide another PVC tube with the length of 40 cm, inner lumen diameter of 5 mm (the tube was named as tubeII) to float selectively into the right lower pulmonary artery under X-ray fluoroscopic monitoring. Then, the Swan-Ganz catheter was extracted out from the inside of the tubeII and the three segmental autologous cylinder blood clots induced ex vivo with 7 cm long and 4 mm wide were selectively injected into the right lower pulmonary lobar artery through the PVC tubeII lumen. Two weeks later, the 18 canines were subdivided into 3 groups. The Ischemia group (Group 2, n = 6) underwent identical surgical procedures except for embolectomy and were observed for 6 hours. The Reperfusion group (Group 3, n = 6) underwent embolectomy for reperfusion in the right lower artery and were observed for 6 hours after embolectomy. The Inhaled NO group (Group 4, n = 6) underwent a process similar to the reperfusion group but with additional administration of inhaled NO at 20 ppm for 6 hours through mechanical ventilation.

### Preparing for four experimental PTE canine models and embolectomy

#### Preparing the animal models before embolectomy

Two weeks after embolization, the PTE canine was anesthetized with 5 ml of intravenous propofol, intraperitoneal injection of 0.5 ml/kg dosage of 3% sodium pentobarbital. Endotracheal intubation followed and they were subsequently connected to Servo900C (SIEMENS company, Germany) with volume controlled ventilation (VCV), tidal volume of 15 ml/kg, inspired oxygen concentration of 40%, respiratory rates of 20 breaths/min, inspiratory time of 25%, inspiratory pause time of 10% and positive end-expiratory pressure (PEEP) of 3 cmH2O. A 7-F double-lumen Swan-Ganz thermistor catheter (Edwards Swan-Ganz, Baxter Healthcare Corporation, Deerfield, IL) was introduced into the pulmonary artery through the left external jugular vein. The Swan-Ganz was connected to a pressure transducer and a multi-channel signal analysis system (Shanghai Alcott Biotech, China) for direct measurement of cardiac output (CO) by thermodilution, mean pulmonary arterial pressure (mPAP), pulmonary artery wedge pressure (PAWP). Pulmonary vascular resistance was calculated accordingly (PVR = (mPAP-PAWP)/CO) × 80). A catheter was inserted into the left femoral artery for direct measurement of mean arterial blood pressure (mBP) and heart rate (HR), and for periodic collection of arterial blood for analysis of arterial PaO2/FiO2, oxygenation index (OI), carbon dioxide and pH.

#### Embolectomy for reperfusion and mechanical ventilation with inhaled NO

A right thoracotomy was performed through the fifth intercostal space. The right lower pulmonary lobe was mobilized by dividing the pulmonary ligament and the hilar structures were then dissected free. By clamping the right lower pulmonary artery hilum, Fogarty arterial embolectomy was performed according to the exact location of the thrombus as we had previously known. Anastomosis of the lower pulmonary artery was performed with non-absorbable 5–0 running sutures. Then, the lower pulmonary artery was unclamped and observed for reperfusion changes for 6 hours.

In the Inhaled NO group, NO delivery device (SensorNOx, SensorMedics Co. Yorba Linda, CA) was introduced into the downstream of the humidifier through the inspiratory limb of the respiratory circuit after the embolectomy. NO was administered at a concentration of 20 ppm, starting immediately after initiating reperfusion and continuing for 6 hours during the reperfusion period. The concentrations of NO and NO2 were determined in a continuous fashion by the SensorNOx delivery device, using electrochemical cell analysis. NO2 levels did not exceed 3 ppm.

A chest tube was inserted and the thoracotomy closed. Intravenous injection of 100U/kg heparin was performed after every surgical procedure. Hemodynamic parameters were measured at baseline (at the time point of 0 hour) and at the time points of 2, 4 and 6 hours after surgical procedures. Each animal was covered during the experimental period to prevent hypothermia.

#### The animals were killed by exsanguination

The lung was removed from each animal for observation. The lung distal the clot were then fixed in 10% formalin and examined accordingly.

**Masson’s trichrome stain** The formalin-fixed lung tissues were embedded in paraffin, cut into 4-mm-thick tissue slices and were stained by Masson’s trichrome according to the instructions of the company (Sigma-aldrich Shanghai Company, USA).

**TUNEL** TUNEL was done according to the manufacturer’s instructions (R&D Systems China (Shanghai) Company, USA) for detection of apoptosis. The slides were analyzed by a blinded pulmonary pathologist.

TUNEL-positive pneumocytes were counted at 400’ in 100 microscopic fields per lung lobe. Only the cells lining the alveolar wall with positive nuclear and no cytoplasmic staining were regarded as apoptotic pneumocytes. Those found within the interstitium or in the alveoli were not counted.

**Ultrastructure** Small pieces of fresh tissue were immersed in universal fixative (1% glutaryl-aldehyde, 4% paraformaldehyde, pH 7.4) immediately after biopsy, post fixed in 2% osmium tetroxide, dehydrated in graded acetones, and embedded in an Epon-Araldite mixture (Fisher Scientific Corp., Toronto, Ontario, Canada). Selected blocks were thin-sectioned, mounted on copper grids, and contrasted with uranyl acetate and lead citrate. The grids were examined for pneumocytes in a Philips 208 s electron microscope (N.V. Philips, Gloeilampenfarbrieken, Eindhoven, Netherlands).

### Statistical analysis

SPSS 11.0 software was used in statistical analysis. Numerical parameters with normal Gaussian distribution (According to Kolmogorov-Smirnov test) were expressed as mean ± 1 standard deviation $\left(\bar{x}\pm s\right)$. The differences of measured parameters among different time point after surgery within same group were analyzed by Repeated Measures ANOVA and the differences between groups by ANOVA. Pearson Correlation Coefficient was used for analyzing the correlation between two variables. *P* value <0.05 was considered as significant differences.

## Abbreviations

LIRI: Lung ischemia–reperfusion injury; NO: Nitric oxide; PTE: Pulmonary thromboembolism; BPA: Balloon pulmonary angioplasty; CTEPH: Chronic thromboembolic pulmonary hypertension; RPE: Reperfusion pulmonary edema; ICU: Intensive care unit; PVC: Polyvinyl chloride; VCV: Volume controlled ventilation; PEEP: Positive end-expiratory pressure; CO: Cardiac output; mPAP: Mean pulmonary arterial pressure; PAWP: Pulmonary artery wedge pressure; mBP: Mean arterial blood pressure; HR: Heart rate; OI: Oxygenation index; BALF: Bronchoalveolar lavage fluid; W/D ratio: Wet to dry ratio; NOS: Nitric oxide synthetase; ROS: Reactive oxygen species; iNOS: Inducible nitric oxide synthase.

## Competing interests

The authors declare that they have no competing interest.

## Authors’ contributions

All authors participated in the design, interpretation of the studies and analysis of the data and review of the manuscript; CD made a substantial contribution to the concept and conducted the experiments, acquisition of data; YY and QL revised the paper for some intellectual content; MY, ZZ, HD, XC and Z H help establish the animal model, prepare for the cylinder autologous blood clots; LZ and JZ recorded the parameters and stained the specimens; KL performed the statistical analysis. All authors read and approved the final manuscript.
